# Efficacy of Hyaluronan-Enriched Transfer Medium for Low-Grade Frozen Embryo Transfer

**DOI:** 10.7759/cureus.39210

**Published:** 2023-05-19

**Authors:** Yoriko Horiuchi, Atsushi Yanaihara, Shota Hatakeyama, Yukino Taga, Tetsurou Kondou, Goro Kuramoto, Shirei Ohgi

**Affiliations:** 1 Obstetrics and Gynecology, Yanaihara Women's Clinic, Kamakura, JPN; 2 Obstetrics and Gynecology, Showa University Koto Toyosu Hospital, Tokyo, JPN; 3 Obstetrics and Gynecology, Tokyo Women's Medical University, Tokyo, JPN

**Keywords:** cd44, gardner classification, assisted hatching, frozen embryo transfer, hyaluronan-enriched transfer medium

## Abstract

Research question: This study aimed to retrospectively evaluate the efficacy of a hyaluronan-enriched transfer medium (HETM) for transfer failures and transfer of frozen embryos that have been graded as C at the time of transfer according to the Gardner classification of trophectoderm (TE).

Design: This study included 365 cycles of unsuccessful frozen-thawed embryo transfers in hormone replacement cycles graded C according to the Gardner classification of TE at the time of transfer. Clinical pregnancy rates were compared using the χ^2^ test, with the patients divided into two groups: one whose transfers did include HETM (HETM group) and one whose transfers did not include HETM (control group). As a subgroup analysis, patients with a TE grade of C at the time of transplantation were divided into two groups: those aged 39 years or younger and those aged 40 years or older at the time of transplantation. The clinical pregnancy rates of the groups with and without HETM were then compared.

Results: No difference in the clinical pregnancy rates between the HETM and control groups was observed.

Conclusions: Hyaluronic acid is believed to favor implantation by promoting adhesion between the embryo and the endometrium, and there are reports of improved implantation and pregnancy rates as a result of HETM. However, the present results suggest limited effectiveness for HETM. Further case series should be conducted, and the suitability of its use as a treatment should be investigated.

## Introduction

Hyaluronic acid is a macromolecular substance that is widely distributed in vivo, is specifically present in the follicular fluid and endometrium, and is thought to play an important role during implantation in assisted reproductive technology. Hyaluronic acid also indirectly promotes vascularization and adhesion between cells as well as cells and substrates; thus, embryos expressing hyaluronic acid receptors and the endometrium are also thought to adhere more easily via hyaluronic acid [1]. In addition, following implantation, the adhesive properties of hyaluronic acid at high concentrations assist the implantation of the embryo [2,3]. The receptor for hyaluronic acid, CD44, is expressed in both the embryo and endometrial stroma, with the highest expression observed during implantation when the endometrium is most receptive [4].

Based on these mechanisms, it is thought that hyaluronan-enriched transfer medium (HETM) may improve the implantation rate of embryos via CD44 expression in the embryo and endometrium, making it an effective infertility treatment. Several randomized controlled trials on the use of HETM in embryo transfer have proposed its potential to improve implantation, pregnancy, and birth rates. The latest report summarizing randomized controlled trials published through 2020 also showed its usefulness [5]. Another report showed significant improvement in clinical pregnancy rate with the use of HETM for fresh embryo transfers (day 3 or day 5 embryos) for cases of repeated failures and in low-grade blastocysts for patients over 35 years of age [6]. This report suggests that HETM affects embryo growth and crosstalk between the embryo and endometrium rather than in the endometrium alone.

However, on the other hand, some reports show no significant differences in implantation, pregnancy, or birth rates with the use of HETM [7,8]. In particular, recent reports have found no significant differences in birth rates with the use of HETM in frozen-thawed embryo transfers [9].

However, as far as we have been able to ascertain, there have been no reports of limited use of HETM with the grading of blastocysts evaluating its efficacy. Therefore, for this study, we opted to limit the use of HETM to low-grade blastocysts, defined as grade C of the Gardner classification of trophectoderm (TE), with the rationale that HETM is suggested to be highly effective in this group. Sparse trophic ectoderm cells are quite detrimental to implantation, and HETM has shown suitability for assisting with this.

Another motivation for this study was that in April of 2022, insurance coverage for infertility treatment including the use of HETM began in Japan. This change is expected to lead to the more frequent use of HETM for the sake of cost-effectiveness. Therefore, we analyzed the results of the use of HETM at our institution for cases of failed transfers and low-grade blastocysts.

## Materials and methods

Study design and patients

A retrospective cohort study was conducted with 332 patients who had grade C TE according to the Gardner classification at the time of transfer, underwent freeze-thaw embryo transfer, and assisted hatching during 365 hormone replacement cycles between 11th October 2014 and 27th April 2022 at our institution.

We compared the clinical pregnancy rates in two groups: one with (HETM group) and one without (control group) the culture medium containing HETM (Vitrolife EmbryoGlue, Vitrolife, Sweden) at the time of embryo transfer. The study group that chose to use the culture medium containing HETM had more embryo transfer failures and a higher average number of transfers; therefore, first-time embryo transfer cases were excluded in both groups to ensure that there was no difference in the average number of transfers.

As a sub-analysis, the clinical pregnancy rates of the HETM and control groups were also compared for patients with grade C of the Gardner classification of TE at the time of transfer and those aged 39 years or less and 40 years or more at the time of transfer.

This study was conducted according to the Code of Ethics of the World Medical Association, and all participants provided informed consent. The Yanaihara Women's Clinic Ethics Committee issued approval (22-002). The authors had the ability to identify individual participants during and after data collection.

Ovarian stimulation protocols

The ovarian stimulation method was selected based on the condition of each patient. First, the minimum stimulation method was defined as spontaneous follicular development without pharmaceutical treatment, clomifene citrate, human menopausal gonadotropin (hMG), or recombinant follicle-stimulating hormone (rFSH). Second, mild stimulation was defined as the use of hMG or rFSH preparations in addition to clomifene citrate or letrozole. Third, the hyperstimulation method was defined as the use of a short protocol using gonadotropin-releasing hormone (GnRH) agonists, a long protocol using GnRH agonists, or an ovarian stimulation protocol using GnRH antagonists with hMG or rFSH.

In vitro fertilization (IVF) procedure

Ovum pickup was performed under transvaginal ultrasound guidance, and oocyte fertilization was also performed using conventional IVF or intracytoplasmic sperm injection. Embryos were then cultured until day 5 or 6 and classified according to Gardner classification; only those with grade C of the Gardner classification of TE at the time of transfer were included in the study.

Embryo transfer procedure

Embryo transfer was performed under transabdominal ultrasound guidance. We equilibrated the HETM at 37°C, 5% O_2_, and 6% CO_2_ for at least one hour before administering it. After thawing the blastocyst for five hours and confirming viability, the blastocysts were transferred to 1 mL HETM 2.5 hours before the transfer. A 1-mL syringe that aspirated 0.1-mL handling medium (Multipurpose Handling Medium with Gentamicin [FujiFilm Irvine Scientific, Santa Ana, CA]) was connected to an embryo transfer catheter (Kitazato ET Catheter, Kitazato Corporation, Fuji, Shizuoka, Japan) before the handling medium was drained. The embryo transfer catheter was then filled with the handling medium, and approximately 2.0 mm of air was aspirated from the tip of the catheter. Approximately 7.0 mm of handling medium was then aspirated from the tip of the embryo transfer catheter, and another 2.0 mm of air was aspirated before approximately 7.0 mm of handling medium was aspirated with the blastocyst. Finally, approximately 2.0 mm of air was aspirated.

Outcome measures

Pregnancy was confirmed by measuring β-hCG levels 9-10 days after the embryo transfer. Clinical pregnancy was defined as the presence of at least one gestational sac documented by vaginal ultrasonography approximately one week after a positive β-hCG pregnancy test.

Statistical analysis

All data are expressed as mean ± standard deviation. All statistical analyses were performed using Easy R (EZR) [10], a modified version of R commander designed to add statistical functions frequently used in biostatistics. An unpaired t-test and Mann-Whitney U-test were used to compare the clinical parameters between the study and control groups. Comparisons of the proportion of causes of infertility, ovarian stimulation protocols, IVF procedure, and clinical pregnancy rate between the two groups were then performed using a chi-square test and Fisher's exact test. Statistical significance was set at p < 0.05.

## Results

A total of 365 embryo transfer cycles were analyzed. The HETM group included 227 embryo transfer cycles, and the control group included 138 embryo transfer cycles. The clinical parameters were similar between the study and control groups (Table 1). The mean age, number of previous transplants, endometrium thickness, ovarian stimulation protocols, IVF procedure, and causes of infertility were similar between the study and control groups.

**Table 1:** Patient characteristics HETM: Hyaluronan-enriched transfer medium; ICSI: Intracytoplasmic sperm injection; IVF: In vitro fertilization.

Parameters	HETM group	Control group	p-values
Total no. of embryo transfer cycles	227	138	
Age (years)	38.2 ± 3.8	38.4 ± 3.8	0.59
Endometrial thickness (mm)	9.9 ± 6.5	9.3 ± 1.4	0.61
No. of previous transplants	2.39 ± 1.90	2.29 ± 1.69	0.18
Causes of infertility, n (%)			
Male factor	15 (6.60)	6 (4.35)	0.49
Tubal	1 (0.44)	0 (0)	1.00
Endometriosis	7 (3.08)	4 (2.90)	1.00
Other	2 (0.88)	1 (0.72)	1.00
Unexplained	202 (88.99)	127 (92.03)	0.37
IVF procedure, n (%)			
Conventional IVF	110 (48.46)	69 (50.00)	0.83
ICSI	117 (51.54)	69 (50.00)	0.83
Ovarian stimulation protocols, n (%)			
Minimum stimulation method	8 (3.52)	3 (2.17)	0.55
Mild stimulation method	192 (84.58)	118 (85.51)	0.88
Hyperstimulation method	27 (11.89)	17 (12.32)	1.00

Clinical pregnancy was defined as the presence of a confirmed gestational sac in the uterus. A comparison of clinical pregnancy rates between the two groups showed no significant difference (31.7% vs 30.4%) (p = 0.817).

When clinical pregnancy rates were compared by age at transplantation in a subgroup analysis, there was also no significant difference in clinical pregnancy rates between the two groups when the age was 39 years or younger (39.3% vs 42.1%) (p = 0.77). No significant difference in clinical pregnancy rates between the two groups was observed when the age at transplant was 40 years or older (19.5% vs 16.1%) (p = 0.67) (Table 2).

**Table 2:** Clinical pregnancy rate HETM: Hyaluronan-enriched transfer medium.

Parameters	HETM group	Control group	p-values
Total	72/227 (31.72)	42/138 (30.43)	0.82
Women < 40 years old	55/140 (39.29)	32/76 (42.11)	0.77
Women ≧ 40 years old	17/87 (19.54)	10/62 (16.13)	0.67

## Discussion

This study found no significant difference in the clinical pregnancy rates between the HETM and control groups. Thus, our results are in line with previous reports, which found no significant difference in birth rates with frozen embryo transfers [9]. However, reports of significant differences in birth rates in fresh embryo transfers do exist, with no significant differences in frozen embryo transfers [11,12]. It is difficult to speculate on the reason for the increased birth rate in fresh embryo transfers alone, but one hypothesis is that hyaluronic acid assists in maintaining the embryonic receptivity of the endometrium, which is reduced by the high estrogen state caused by ovarian stimulation during fresh embryo transfer [9].

Although it is unclear why HETM is effective in fresh but not frozen embryo transfers, we hypothesized that the lack of a significant difference in clinical pregnancy rates, at least in our study that was limited to frozen embryo transfers, may be related to endometrial receptivity rather than embryonic receptivity.

A recent report stated that uterine natural killer (NK) cells, immune cells of the endometrium, express CD44, which is also a receptor for hyaluronic acid; further, high-molecular-weight hyaluronic acid inhibits decidualization by uterine NK cells, while low-molecular-weight hyaluronic acid promotes uterine NK cell function, maintains decidualization, and facilitates implantation [13]. Uterine NK cells are immune cells that reside in the endometrium, proliferate after ovulation, and occupy >30% of the endometrial stroma. When the endometrial stroma becomes decidualized, NK cells migrate together and eventually gather at the implantation site. NK cells interact with the trophoblastic cells of the placenta, transforming the uterine helical artery into a blood vessel with high blood flow, ensuring blood supply to the placenta and fetus. However, the functions of NK cells in these processes are not well understood [14].

High-molecular-weight hyaluronic acid may inhibit the maintenance of decidualization, which is the original function of CD44-expressing uterine NK cells. In addition, low-grade blastocysts secrete less hyaluronidase-2, which degrades high-molecular-weight hyaluronan, resulting in high-molecular-weight hyaluronan inhibiting uterine NK cell activity, which may, in turn, promote endometrial to menstrual-like changes [13]. If this is indeed the case, the addition of high-concentration hyaluronic acid is not beneficial for the endometrial environment and is unlikely to improve implantation and pregnancy rates. However, the addition of hyaluronidase-2 in addition to high concentrations of hyaluronic acid may increase uterine NK cell activity owing to low-molecular-weight hyaluronic acid while maintaining a suitable state for implantation in the endometrium.

In the present study, HETM was used in low-grade blastocysts with low hyaluronidase-2 secretion capacity. This may have inhibited uterine NK cell activity, resulting in an endometrial environment unsuitable for implantation. These results are consistent with the previous findings that did not show a significant difference in pregnancy rates between groups that used HETM and those that did not, indicating that high concentrations of hyaluronic acid alone are not favorable as an implantation environment and may not contribute to improved pregnancy rates.

Additionally, it is reported that high concentrations of hyaluronic acid used in vitro did not improve the adhesion of human blastocysts to endometrial cells [15]. These reports support the present study’s finding that HETM does not contribute to improved clinical pregnancy rates. However, it has also been reported that when hyaluronic acid synthesis in the trophic ectoderm of mouse blastocysts is blocked, the implantation rate is reduced both in vitro and in vivo [16]. Based on various reports, it is conceivable that hyaluronic acid may play an important role during implantation. Therefore, it is necessary to determine the optimal concentration of hyaluronic acid (HA).

It should be noted that in the present study, the assessment of the endometrium was based solely on its thickness, which limits its functional assessment. Another limitation is that even in blastocysts, the assessment is gross, and no further examination of the presence or absence of chromosomal abnormalities is possible.

Infertility treatment and the use of HETM at the time of implantation are now covered by insurance in Japan. This is expected to increase the number of cases in which HETM is used in the future regardless of the condition of the embryo or endometrium. However, some add-ons in IVF treatment lack medical evidence and are undesirable from the perspective of cost-effectiveness [17]; there are reports that the use of HETM is also effective in improving implantation and pregnancy rates as well as reports that it is not effective, and thus further large-scale surveys are needed. It will be necessary to select cases in which HETM should be used with consideration of cost-effectiveness.

## Conclusions

This study found no significant difference in the clinical pregnancy rates between the HETM and control groups, which is in line with the findings of some previous reports. However, other pre-existing evidence remains encouraging for continued investigation. Until sufficient evidence is accumulated, the decision to use or not to use HETM should be made after careful consideration of cost-effectiveness and consultation with patients.
